# Relationship between Maternal Body Composition during Pregnancy and Newborn Birth Weight in Japan

**DOI:** 10.31662/jmaj.2025-0060

**Published:** 2025-11-14

**Authors:** Eriko Eto, Masakazu Kato, Satoe Kirino, Chiaki Kuriyama, Shujiro Sakata, Hikari Nakato, Sakurako Mishima, Akiko Ohira, Hisashi Masuyama

**Affiliations:** 1 Department of Obstetrics and Gynecology, Okayama University Graduate School of Medicine, Dentistry, and Pharmaceutical Sciences, Okayama, Japan

**Keywords:** maternal body composition, newborn birth weight, pre-pregnancy body mass index, fat-free mass, fat mass gain

## Abstract

**Introduction:**

This study aimed to investigate the changes in maternal body composition during pregnancy in Japanese women and the relationship between maternal body composition and newborn birth weight using pre-pregnancy body mass index (BMI) in all trimesters.

**Methods:**

A total of 1,851 pregnant Japanese women were enrolled in this study. Body composition was measured using TANITA MC-190EM. The associations between newborn birth weight and maternal BMI, fat mass (FM), fat-free mass (FFM), total body water (TBW), muscle mass (MM), FM gain, FFM gain, and weight gain were evaluated.

**Results:**

The participants’ age and pre-pregnancy BMI were 34.1 years and 21.4 kg/m^2^, respectively. Among the patients, 13.4%, 73.0%, 10.3%, and 3.3% were underweight, average weight, overweight, and obese, respectively. The FM showed no significant change from the second to third trimesters in the underweight, overweight, and obese groups. Moreover, the FM in the overweight and obese groups did not change during any period. The FFM, TBW, and MM significantly increased from the first to second and second to third trimesters. In BMI-stratified multivariate regression analyses, FFM in the normal and overweight groups was positively associated with birth weight, whereas FM gain was negatively associated in the underweight and normal groups. No significant associations were observed in the obese group.

**Conclusions:**

Changes in maternal body composition during pregnancy in Japanese women varied by pre-pregnancy BMI. Associations with birth weight also differed by BMI group. Further prospective studies are needed to confirm these relationships and investigate the mechanisms.

## Introduction

Maternal body size and nutritional status are known to be associated with birth weight ^(1), (2), (3), (4), (5)^. Recently, the relationship between maternal body composition and newborn birth weight has attracted the attention of researchers, owing to the availability of simple and accurate body composition measurements. Body fat mass (FM), fat-free mass (FFM), total body water (TBW), and muscle mass (MM) can be calculated using Bioelectrical Impedance Analysis (BIA) to accurately reflect body composition and are considered better predictors of maternal nutritional status than body mass index (BMI) ^(6), (7)^.

Although the relationship between maternal body composition and newborn birth weight has been investigated, the results have been consistent. Some studies have shown a correlation with FFM ^(8), (9), (10)^; however, the reason increased FFM is associated with high newborn birth weight remains unclear. Moreover, the body composition varies by race ^(11), (12)^. To the best of our knowledge, no previous study has clarified the relationship between maternal body composition and birth weight in pregnant Japanese women, and no reports exist regarding the differences in the predictors of newborn birth weight based on pre-pregnancy BMI.

The maternal nutritional status and changes in body composition during pregnancy may vary with pre-pregnancy BMI. Detailed body composition studies may define new nutritional therapy methods because current therapy during pregnancy focuses only on weight gain (WG) guidance based on pre-pregnancy BMI. The updated Institute of Medicine (IOM) guidelines incorporated the World Health Organization categories of maternal BMI; however, they were based on lower general population BMI with limited ethnic diversity. Although the American College of Obstetricians and Gynecology endorsed these guidelines, they have not been universally implemented ^(13), (14)^

Therefore, in this retrospective study, we aimed to reveal the changes in maternal body composition during pregnancy in Japanese women. In addition, we investigated the relationship between maternal body composition and newborn birth weight on the basis of pre-pregnancy BMI in the first, second, and third trimesters.

## Materials and Methods

### Study design

This was a retrospective cohort study in patients who gave birth at Okayama University Hospital. The study was conducted in accordance with the Declaration of Helsinki and the Ethical Guidelines for Medical and Health Research Involving Human Subjects in Japan. Data were obtained from routine antenatal care records. The study protocol was approved by the Ethics Committee of Okayama University Graduate School of Medicine, Dentistry, and Pharmaceutical Sciences and Okayama University Hospital. Because this was a retrospective study using anonymized clinical data, written informed consent was waived by the ethics committee. Instead, study information was disclosed on the institutional website, and participants were given the opportunity to opt out, in accordance with the approved protocol.

### Participants and procedures

**Figure 1.**
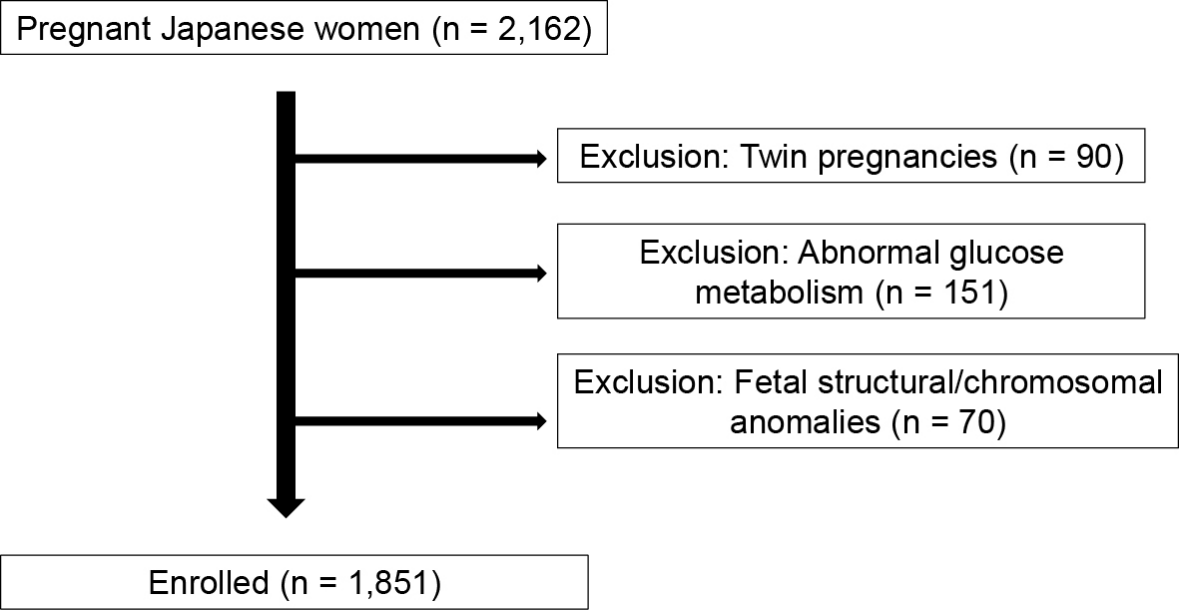
Flowchart of study participant selection.

Maternal body composition was measured in all pregnant women at each prenatal checkup. This study was limited to pregnant Japanese women, who had given birth from 37 to 41 weeks of pregnancy. Multiple pregnancies, pregnant women with abnormal glucose metabolism, and fetal structural anomalies or chromosomal abnormalities were excluded. A flowchart of study participant selection is shown in Figure 1.

### Measurements

Body composition was measured during each trimester using a foot-to-foot BIA system (TANITA MC-190EM; TANITA, Tokyo, Japan). The participants stood erect on the footpads of the analyzer with bare feet, and measurements were obtained when the grips were held. Electric current was supplied from electrodes placed on the tips of the toes and fingers, and the machine measured the voltage on the heels of both feet and near the sides of both hands. Weight, muscle volume, FM, FFM, TBW, and MM were analyzed. We used the maternity mode to correct the weight of the fetus according to the gestational age. Several studies have been conducted to determine the accuracy and reliability of the BIA device ^(15), (16), (17)^. Data on maternal age, pre-pregnancy BMI, weeks and mode of delivery, and newborn birth weight were collected from the medical records.

### Statistical analysis

Differences among the BIA measures of FM, FFM, TBW, and MM as mean continuous variables were evaluated using analysis of variance with a standard Tukey honestly significant difference adjustment for multiple comparisons. Univariable correlations of newborn birth weight with maternal FM, FFM, WG, FM gain, FFM gain, and pre-pregnancy BMI were assessed using Pearson correlation coefficients according to the distribution to identify the predictors of newborn birth weight. Variables identified as having a statistically significant relationship with newborn birth weight were incorporated into a multivariable linear regression model, with newborn birth weight as the dependent variable. Moreover, stratified analyses were conducted by pre-pregnancy BMI categories: underweight (<18.5), normal (18.5-24.9), overweight (25.0-29.9), and obese (≥30). Multivariable regression models were adjusted for gestational age at delivery, infant sex, and hypertensive disorders of pregnancy. Statistical significance was set at p < 0.05. The analyses were performed using BellCurve for Excel version 3.21.

## Results

A total of 2,162 pregnant women were registered during the study period. After excluding 90 women with multiple pregnancies, 151 women with abnormal glucose metabolism (including gestational diabetes mellitus and pre-existing diabetes), and 70 women with fetal anomalies or chromosomal abnormalities, 1,851 women were included in the final analysis. The body composition measurements were obtained for 801, 1,055, and 1,436 women during the first, second, and third trimesters, respectively. The characteristics of the study population are summarized in Table 1. The mean age was 34.1 years, and the mean pre-pregnancy BMI was 21.4 kg/m^2^. The evaluation of these measurements revealed that 13.4% were underweight; 73.0% had an average weight; 10.3% were overweight, and 3.3% were obese.

**Table 1.** Characteristics of the Study Population.

Parameter	Value
Age (years)	34.1 (16-46)
Primiparous	55.1
Pre-pregnancy BMI (kg/m^2^)	21.4 (15.1-42.1)
BMI category (kg/m^2^)	
Underweight (<18.5)	13.4
Normal (18.5-24.9)	73.0
Overweight (25.0-29.9)	10.3
Obese (30 or higher)	3.3
Gestation age at delivery (weeks)	38.8 (37-42)
Mode of delivery (%)	
Spontaneous vaginal delivery	61.1
Instrumental	7.1
Cesarean delivery	31.8
Newborn birth weight (kg)	3.0 (1.7-4.7)

BMI: body mass index. Data are presented as means (ranges) or percentages unless otherwise specified.

The maternal body composition in each trimester is presented in Table 2. The maternal body composition in each trimester according to the pre-pregnancy BMI is presented in Table 3. Supplementary Figures 1-4 illustrate the changes in the FM, FFM, TBW, and MM during the first to third trimester in each pre-pregnancy BMI category. The FM showed no significant differences from the second to third trimesters in the underweight, overweight, and obese groups. Moreover, the FM in the overweight and obese groups did not change during any period. The FFM, TBW, and MM significantly increased from the first to second and second to third trimesters.

**Table 2.** Maternal Body Composition.

	First trimester (n = 801)	Second trimester (n = 1,055)	Third trimester (n = 1,436)
FM (kg)	15.0 (4.3-38.3)	16.7 (6.2-39.1)	16.9 (5.7-38.5)
FFM (kg)	37.9 (29.1-49.5)	39.3 (29.3-52.1)	40.4 (30.7-53.9)
TBW (kg)	26.2 (18.7-36.2)	27.6 (19.4-40.3)	29.0 (20.1-49.7)
MM (kg)	35.7 (27.7-46.3)	37.2 (27.9-51.1)	38.3 (29.2-52.6)
FM gain (kg)	-	1.6 (−11.0 to 8.6)	1.7 (−11.0 to 9.3)
FFM gain (kg)	-	1.4 (−7.2 to 11.3)	2.7 (−7.5 to 12.0)
WG (kg)	-	5.0 (−5.1 to 14.9)	8.5 (−3.6 to 20.5)

FFM: fat-free mass; FM: fat mass; MM: muscle mass; TBW: total body water; WG: weight gain. FM, FFM, and WG are shown by the increase from the first trimester. Data are presented as means (ranges).

**Table 3.** Maternal Body Composition Based on the Pre-Pregnancy BMI.

Underweight	First trimester (n = 115)	Second trimester (n = 114)	Third trimester (n = 212)
Pre-pregnancy BMI	17.6 (15.5-18.4)	17.6 (15.5-18.4)	17.6 (15.5-18.4)
FM (kg)	8.8 (4.3-16.0)	11.2 (6.2-16.2)	11.8 (5.7-17)
FFM (kg)	35.5 (29.1-42.1)	37.4 (30.2-42.7)	38.2 (30.7-45.4)
TBW (kg)	23.6 (18.7-28.3)	25.3 (19.5-31.2)	26.4 (20.2-33.5)
MM (kg)	33.5 (27.7-39.6)	35.3 (28.7-40.1)	36.1 (29.2-42.6)
FM gain (kg)	-	1.9 (−6.9 to 7.2)	2.4 (−8.0 to 7.9)
FFM gain (kg)	-	1.5 (−5.3 to 11.3)	2.5 (−4.5 to 11.6)
WG (kg)	-	6.1 (−0.2 to 11.9)	9.4 (0.8-16.4)
NBW (kg)	2.9 (1.8-4.3)	2.9 (1.8-4.3)	2.9 (1.8-4.3)
**Normal weight**	**First trimester (n = 595)**	**Second trimester (n = 781)**	**Third trimester (n = 1027)**
Pre-pregnancy BMI	20.9 (18.5-24.9)	20.9 (18.5-24.9)	20.9 (18.5-24.9)
FM (kg)	14.4 (7.2-25.5)	16.2 (7.6-29.7)	16.6 (7.8-30.5)
FFM (kg)	38.0 (29.1-47.7)	39.2 (29.3-54.7)	40.4 (30.8-56.3)
TBW (kg)	26.2 (19.7-35.5)	27.5 (20.7-39.0)	28.7 (21.4-45.1)
MM (kg)	35.8 (27.7-44.7)	36.9 (27.9-51.1)	38.0 (29.3-52.6)
FM gain (kg)	-	1.8 (−6.9 to 7.2)	2.2 (−8.0 to 9.5)
FFM gain (kg)	-	1.4 (−5.3 to 11.3)	2.5 (−4.5 to 11.6)
WG (kg)	-	5.1 (−5.1 to 17.1)	8.6 (−9.9 to 18.7)
NBW (kg)	3.0 (1.8-4.4)	3.0 (1.8-4.4)	3.0 (1.8-4.4)
**Overweight**	**First trimester (n = 76)**	**Second trimester (n = 120)**	**Third trimester (n = 144)**
Pre-pregnancy BMI	27.0 (25.0-29.9)	27.0 (25.0-29.9)	27.0 (25.0-29.9)
FM (kg)	25.1 (16.4-33.9)	25.7 (14.6-37.8)	25.0 (13.7-36.1)
FFM (kg)	39.8 (32.5-49.5)	42.3 (33.8-57.8)	44.0 (34.7-61.0)
TBW (kg)	29.1 (24.3-36.2)	31.1 (24.8-40.3)	32.6 (25.7-42.1)
MM (kg)	37.5 (30.8-46.3)	39.8 (32.0-53.9)	41.4 (32.8-56.8)
FM gain (kg)	-	1.0 (−6.9 to 7.2)	−0.95 (−10.2 to 7.9)
FFM gain (kg)	-	1.6 (−5.3 to 11.3)	3.4 (−2.8 to 12.0)
WG (kg)	-	2.7 (−8.0 to 10.9)	5.7 (−7.5 to 15.6)
NBW (kg)	3.1 (2.0-4.4)	3.1 (2.0-4.4)	3.1 (2.0-4.4)
**Obese**	**First trimester (n = 15)**	**Second trimester (n = 40)**	**Third trimester (n = 53)**
Pre-pregnancy BMI	33.9 (30.0-45.0)	33.9 (30.0-45.0)	33.9 (30.0-45.0)
FM (kg)	34.6 (27.8-38.3)	38.6 (25.5-69.0)	36.3 (21.6-64.7)
FFM (kg)	41.1 (35.9-47.5)	44.7 (34.5-58.5)	46.8 (36.9-63.3)
TBW (kg)	31.6 (28.7-35.2)	34.7 (27.5-45.8)	36.0 (29.5-49.7)
MM (kg)	38.6 (33.9-44.5)	42.0 (32.5-54.5)	43.9 (34.9-59.0)
FM gain (kg)	-	1.3 (−11.0 to 7.3)	1.4 (−11.0 to 7.9)
FFM gain (kg)	-	1.3 (−7.2 to 11.3)	2.5 (−7.5 to 11.9)
WG (kg)	-	1.4 (−10.1 to 8.9)	4.4 (−8.9 to 13.4)
NBW (kg)	3.2 (2.1-5.2)	3.2 (2.1-5.2)	3.2 (2.1-5.2)

BMI: body mass index; FFM: fat-free mass; FM: fat mass; MM: muscle mass; NBW: newborn birth weight; TBW: total body water; WG: weight gain. FM, FFM, and WG are shown by the increase from the first trimester. Data are presented as means (ranges).

The associations between newborn birth weight and maternal BMI, FM, FFM, FM gain, FFM gain, and WG were evaluated. In the univariable analysis, the maternal FFM and FM gain in almost every second and third trimester showed the strongest correlation with newborn birth weight (Table 4). Table 5 lists the results of multivariable linear regression analyses in the third trimester, stratified by maternal BMI category. In the underweight group, FM gain was negatively associated with birth weight (β = −35.44, 95% confidence interval [CI]: −68.1 to −2.78, p = 0.0337), whereas FFM and GWG were not significant predictors. In the normal BMI group, FFM showed a significant positive association with birth weight (β = 19.92, 95% CI: 10.78-29.05, p < 0.0001), and FM gain was negatively associated (β = −18.37, 95% CI: −32.36 to −4.37, p = 0.0102). In the overweight group, FFM was positively associated with birth weight (β = 30.38, 95% CI: 7.98-52.77, p = 0.0087), whereas FM gain and GWG were not significantly associated. In the obese group, none of the body composition parameters were significantly associated with birth weight.

**Table 4.** Univariate Analysis Regarding the Correlations with Newborn Birth Weight Based on the Pre-Pregnancy BMI.

Underweight	First trimester (n = 115)	Second trimester (n = 114)	Third trimester (n = 212)
Pre-pregnancy BMI	0.120	0.22	0.09
FM (kg)	0.20	0.22	0.27
FFM (kg)	0.23	0.32***	0.35***
FM gain (kg)	-	0.13*	0.17
FFM gain (kg)	-	0.06	0.19*
WG (kg)	-	0.15	0.17
**Normal weight**	**First trimester (n = 595)**	**Second trimester (n = 781)**	**Third trimester (n = 1027)**
Pre-pregnancy BMI	0.09	0.13	0.13
FM (kg)	0.11	0.36	0.22
FFM (kg)	0.20***	0.26***	0.23**
FM gain (kg)	-	0.27**	0.12*
FFM gain (kg)	-	0.07	0.08
WG (kg)	-	0.30	0.14
**Overweight**	**First trimester (n = 76)**	**Second trimester (n = 120)**	**Third trimester (n = 144)**
Pre-pregnancy BMI	0.10	0.22	0.17
FM (kg)	0.10	0.24	0.41***
FFM (kg)	0.10	0.30**	0.24
FM gain (kg)	-	0.07	0.10
FFM gain (kg)	-	0.12	0.13
WG (kg)	-	0.17**	0.31
**Obese**	**First trimester (n = 15)**	**Second trimester (n = 40)**	**Third trimester (n = 53)**
Pre-pregnancy BMI	0.04	0.06	0.23
FM (kg)	-0.14	0.02	0.22
FFM (kg)	0.29	0.40*	0.36***
FM gain (kg)	-	−0.001	0.05**
FFM gain (kg)	-	0.11	0.16
WG (kg)	-	0.09	0.13

BMI: body mass index; FFM: fat-free mass; FM: fat mass; NBW: newborn birth weight; WG: weight gain. FM, FFM, and WG are shown by the increase from the first trimester. * p < 0.05 ** p < 0.01 *** p < 0.001 Data are presented as correlation coefficients.

**Table 5.** Multivariate Linear Regression for Newborn Birth Weight in the Third Trimester (Adjusted Model).

BMI category	FFM (β, 95% CI)	p	FM gain (β, 95% CI)	p	WG (β, 95% CI)	p
Underweight	14.81 (−7.94 to 37.57)	0.1995	−35.44 (−68.1 to −2.78)	0.0337	0.79 (−18.5 to 20.09)	0.9351
Normal	19.92 (10.78 to 29.05)	<0.0001	−18.37 (−32.36 to −4.37)	0.0102	−2.35 (−12.09 to 7.4)	0.6365
Overweight	30.38 (7.98 to 52.77)	0.0087	−17.23 (−46.55 to 12.09)	0.2449	7.08 (−15.46 to 29.62)	0.5325
Obese	−1.45 (−38.11 to 35.2)	0.9335	−2.95 (−34.64 to 28.74)	0.8446	−8.1 (−40.49 to 24.29)	0.6000

CI: confidence interval; BMI: body mass index; FFM: fat-free mass; FM gain: fat mass gain; WG, weight gain. FM gain is shown by the increase from the first trimester. Data are presented as the regression coefficients (95% CIs).

## Discussion

In this study, two important clinical observations were made. First, the change in maternal body composition differed on the basis of the pre-pregnancy BMI, particularly for the FM. Second, FFM and FM gain were associated with newborn birth weight in third trimester.

The change in maternal body composition differed on the basis of the pre-pregnancy BMI, especially regarding the FM. Hytten17 reported that 3-5 kg of FM is stored during pregnancy and that a total storage requirement of 30,000 kcal is needed. The FM initially increases rapidly but gradually levels off in the third trimester. In our study, we showed this using the BIA method. When comparing pre-pregnancy BMIs, no increase was found in FM from the first trimester in pregnant women with overweight and those with obesity; furthermore, FM did not change throughout pregnancy. In contrast, the FFM, TBW, and MM continued to increase until the third trimester, regardless of the pre-pregnancy BMI. In this study, we showed for what we believe is the first time that changes in body composition differed depending on the pre-pregnancy BMI of the pregnant women. The FM increased from the first to the second trimester and did not change in women with underweight from the second to the third trimester. However, the FM did not change during pregnancy in women with overweight and those with obesity. A meta-analysis reported that maternal pre-pregnancy BMI and WG were associated with the risk of pregnancy complications ^(18)^. Mothers with obesity with high WG are at the highest risk of pregnancy complications. Promoting healthy pre-pregnancy BMI and WG may reduce the burden of pregnancy complications and ultimately the risk of maternal and neonatal morbidities. Another meta-analysis proposed that maternal BMI and WG may be associated with the risk of cancer in the offspring ^(19)^. Our findings provide evidence for a relationship between WG and FM gain, particularly in women with overweight or those with obesity. Detailed medical and nutritional therapy can be determined through individual changes in maternal body composition.

Second, FFM and FM gain were associated with newborn birth weight in third trimester. No studies have evaluated the association between newborn birth weight and body composition according to the maternal pre-pregnancy BMI. We found FFM to be a significant predictor regardless of the BMI category. Moreover, FM gain from the first trimester was found to be a significant predictor of newborn birth weight in the second and third trimesters for the underweight and normal weight groups and in the third trimester for the overweight and obese groups. The effects of body composition on pregnant adolescents have been reported, and adequate medical nutrient therapy is essential ^(16), (20)^. Currently, nutritional therapy is based solely on optimal WG, calculated using the mother’s pre-pregnancy BMI. In the United States, weight recommendations for the gestational period are provided by the IOM, which aims for a birth weight of 3,000-4,000 g at 39-40 weeks of gestation. Our subgroup analysis by maternal BMI category revealed distinct patterns in the associations between maternal body composition and newborn birth weight. In the normal and overweight groups, FFM during the third trimester was positively associated with birth weight, suggesting the importance of maternal lean mass in supporting fetal growth in these categories. Interestingly, in the underweight or normal group, FM gain showed a significant negative association with newborn birth weight. From a clinical perspective, this finding might highlight the need for individualized nutritional strategies. Rather than focusing solely on overall WG, FFM and FM could be important factors for medical nutrition therapy for pregnant women. In contrast, in the obese group, none of the body composition components were significantly associated with newborn birth weight, possibly owing to underlying metabolic or placental factors that attenuate maternal-fetal nutrient transfer. These findings highlight the heterogeneous was associated with of maternal body composition on fetal growth depending on pre-pregnancy BMI.

Although the magnitude of the association between maternal FFM gain and neonatal birth weight was modest, even small shifts in birth weight distributions can have meaningful implications at the population level. In the context of prenatal nutritional counseling, such associations may help refine individualized strategies to optimize fetal growth and long-term health outcomes.

Recently, many large-scale studies have been published evaluating the risk of WG during pregnancy in Asian countries, including Japan, using IOM standards. These studies concluded that the IOM standards can be adapted to the Asian population ^(21), (22), (23)^. Our results showed that the FM did not change until the third trimester. However, excessive FM gain can cause higher newborn birth weight. This study can aid in the development of new nutritional therapies based on FM gain in mid- and late-term pregnancies.

Kent et al. ^(10)^ reported that newborn birth weight was positively associated with maternal FFM during the first trimester of pregnancy. Wang et al. ^(24)^ revealed that changes in maternal FFM during pregnancy independently was associated with birth weight. Furthermore, Gernand et al. ^(25)^ reported that high maternal FFM at 0-10 weeks of gestation was independently associated with increased birth weight. In contrast, Forsum et al. ^(26)^ reported that birth weight was correlated with maternal FM content both before pregnancy and at 32 weeks of gestation. However, this conclusion is controversial owing to the small sample size, ethnicity, and testing periods used in previous studies. Our study used the BIA method with a large sample size and maternity mode in which the intrauterine fetal components (fetus, placenta, and amniotic fluid) were modified, which we believe provided a more accurate measurement of the maternal body composition. However, the reason the FFM correlated with newborn birth weight remains unclear. The FFM comprises TBW, bones, and proteins. An increase in FFM is primarily due to an increase in TBW. The notable increase in non-adipose tissue was an increase of 6-7 L of water, measured directly. The bone and carbohydrate content increased slightly, and there was a slight increase in bone mass and <1 kg of protein during pregnancy. The increase in non-bone minerals was low ^(27), (28), (29)^. Previous studies have shown that increased TBW is associated with birth weight ^(30), (31), (32), (33)^. TBW is related to neonatal birth weight because the amount of blood supplied to the fetus through the placenta is reduced if the increase in circulating blood volume in a pregnant woman is insufficient, and adversely was associated with fetal development. In addition, decreased TBW and poor average plasma increase may lead to increased blood viscosity and inadequate oxygen supply to the tissues ^(34)^.

This study had three limitations. First, the pre-pregnancy BMI was self-reported in almost all cases. However, the pre-pregnancy BMI was reliable because the BMI data were obtained during early pregnancy, a period of low maternal weight change. The second limitation was the single-center retrospective nature of the study. Owing to the low BMI of Japanese women, the number of obese cases was minimal. Therefore, future multicenter data analyses are required. Third, this was an observational retrospective study; therefore, we cannot infer causal relationships from the observed associations. Unmeasured or residual confounding may also have influenced the results.

To the best of our knowledge, this is the first study on the birth data of pregnant Japanese women. Most previous studies have been conducted only in North American and European countries; therefore, we believe this study is significant for East Asia. Our findings showed that the results of some previous reports can apply to pregnant Japanese women.

In conclusion, maternal body composition changes during pregnancy were associated with newborn birth weight. Further prospective studies are needed to confirm these relationships and investigate the mechanisms.

## Article Information

### Acknowledgments

We thank Editage (www.editage.jp) for English language editing.

### Author Contributions

Eriko Eto: Conceptualization; Data curation; Formal analysis; Investigation; Methods; Project administration; Supervision, Writing - review and editing. Masakazu Kato and Satoe Kirino: Conceptualization; Data curation; Formal Analysis; Investigation; Visualization; Writing - original draft; Writing - review and editing. Chiaki Kuriyama, Shujiro Sakata, Hikari Nakato, Sakurako Mishima, and Akiko Ohira : Conceptualization; Investigation. Hisashi Masuyama: Conceptualization; Project administration; Supervision.

### Conflicts of Interest

None

### Availability of Data and Materials

The data set cannot be published online because it contains details that may violate participants’ privacy. However, it is obtainable on request.

### Ethics Approval and Consent to Participate

This study was conducted according to the guidelines of the Declaration of Helsinki. All procedures involving human subjects/patients were approved by the Ethics Committee of Okayama University Graduate School of Medicine, Dentistry, and Pharmaceutical Sciences and Okayama University Hospital (Number 2509-019).

### Consent for Publication

Consent for publication of this study was obtained from all participants.

## Supplement

Supplementary Material Supplementary Figure 1. Association between maternal body composition changes and birth weight in women with underweight.
FFM: fat-free mass; FM: fat mass; MM: muscle mass TBW: total body water.
* p < 0.05, ** p < 0.01
Supplementary Figure 2. Association between maternal body composition changes and birth weight in women with normal weight.
FFM: fat-free mass; FM: fat mass; MM: muscle mass TBW: total body water.
* p < 0.05, ** p < 0.01
Supplementary Figure 3. Association between maternal body composition changes and birth weight in women with overweight.
FFM: fat-free mass; FM: fat mass; MM: muscle mass TBW: total body water.
* p < 0.05, ** p < 0.01
Supplementary Figure 4. Association between maternal body composition changes and birth weight in women with obesity.
FM FFM: fat-free mass; FM: fat mass; MM: muscle mass TBW: total body water.
* p < 0.05, ** p < 0.01

## References

[1] Goldstein RF, Abell SK, Ranasinha S, et al. Association of gestational weight gain with maternal and infant outcomes: a systematic review and meta-analysis. JAMA. 2017;317(21):2207-25. 28586887 10.1001/jama.2017.3635 PMC5815056

[2] Nohr EA, Vaeth M, Baker JL, et al. Combined associations of prepregnancy body mass index and gestational weight gain with the outcome of pregnancy. Am J Clin Nutr. 2008;87(6):1750-9. 18541565 10.1093/ajcn/87.6.1750

[3] Kramer MS. Determinants of low birth weight: methodological assessment and meta-analysis. Bull World Health Organ. 1987;65(5):663-737. 3322602 PMC2491072

[4] Hedderson MM, Gunderson EP, Ferrara A. Gestational weight gain and risk of gestational diabetes mellitus. Obstet Gynecol. 2010;115(3):597-604. 20177292 10.1097/AOG.0b013e3181cfce4f PMC3180899

[5] Scholl TO, Hediger ML, Khoo CS, et al. Maternal weight gain, diet and infant birth weight: correlations during adolescent pregnancy. J Clin Epidemiol. 1991;44(4-5):423-8. 2010786 10.1016/0895-4356(91)90081-j

[6] Hogan JL, Farah N, O’Connor N, et al. Bioelectrical impedance analysis and maternal body composition: reproducibility of bioelectrical impedance when analyzing maternal body composition. Int J Body Comput Res. 2011;9:43-8.

[7] Velazquez-Alva M, Irigoyen-Camacho ME, Huerta-Huerta R, et al. A comparison of dual energy X-ray absorptiometry and two bioelectrical impedance analyzers to measure body fat percentage and fat-free mass index in a group of Mexican young women. Nutr Hosp. 2014;29(5):1038-46. 24951983 10.3305/nh.2014.29.5.7254

[8] Sanin Aguirre LH, Reza-López S, Levario-Carrillo M. Relation between maternal body composition and birth weight. Biol Neonate. 2004;86(1):55-62. 15057023 10.1159/000077586

[9] Villar J, Cogswell M, Kestler E, et al. Effect of fat and fat-free mass deposition during pregnancy on birth weight. Am J Obstet Gynecol. 1992;167(5):1344-52. 1442988 10.1016/s0002-9378(11)91714-1

[10] Kent E, O’Dwyer V, Fattah C, et al. Correlation between birth weight and maternal body composition. Obstet Gynecol. 2013;121(1):46-50. 23232753 10.1097/aog.0b013e31827a0052

[11] Widen EM, Gallagher D. Body composition changes in pregnancy: measurement, predictors and outcomes. Eur J Clin Nutr. 2014;68(6):643-52. 24667754 10.1038/ejcn.2014.40 PMC4078736

[12] Wulan SN, Westerterp KR, Plasqui G. Ethnic differences in body composition and the associated metabolic profile: a comparative study between Asians and Caucasians. Maturitas. 2010;65(4):315-9. 20079586 10.1016/j.maturitas.2009.12.012

[13] Physical status: the use and interpretation of anthropometry. Report of a WHO expert committee. World Health Organ Tech Rep Ser. 1995;854:1-452. 8594834

[14] Weight gain during pregnancy: committee opinion 548. Committee on Obstetric Practice, American College of Obstetricians and Gynecologists. 2013 [cited 2022 Aug 14]. Available from https://www.acog.org/-/media/Committee-Opinions/Committee-on-Obstetric-Practice/co548.pdf?dmc=1&ts=20160723T0702247216

[15] Yu OK, Rhee YK, Park TS, et al. Comparisons of obesity assessments in over-weight elementary students using anthropometry, BIA, CT and DEXA. Nutr Res Pract. 2010;4(2):128-35. 20461201 10.4162/nrp.2010.4.2.128 PMC2867223

[16] Lukaski HC, Siders WA, Nielsen EJ, et al. Total body water in pregnancy: assessment by using bioelectrical impedance. Am J Clin Nutr. 1994;59(3):578-85. 8116533 10.1093/ajcn/59.3.578

[17] Hytten FE. Nutrition in pregnancy. Postgrad Med J. 1979;55(643):295-302. 382160 10.1136/pgmj.55.643.295 PMC2425462

[18] Santos S, Voerman E, Amiano P, et al. Impact of maternal body mass index and gestational weight gain on pregnancy complications: an individual participant data meta-analysis of European, North American and Australian cohorts. BJOG. 2019;126(8):984-95. 30786138 10.1111/1471-0528.15661 PMC6554069

[19] Miao J, Chen Y, Liu X, et al. Maternal body mass index, gestational weight gain, and risk of cancer in offspring: a systematic review and meta-analysis. Nutrients. 2023;15(7):1601. 37049442 10.3390/nu15071601 PMC10096488

[20] Thame M, Trotman H, Osmond C, et al. Body composition in pregnancies of adolescents and mature women and the relationship to birth anthropometry. Eur J Clin Nutr. 2007;61(1):47-53. 16835598 10.1038/sj.ejcn.1602484

[21] Enomoto K, Aoki S, Toma R, et al. Pregnancy outcomes based on pre-pregnancy body mass index in Japanese women. PLoS One. 2016;11(6):e0157081. 27280958 10.1371/journal.pone.0157081 PMC4900523

[22] Li C, Liu Y, Zhang W. Joint and independent associations of gestational weight gain and pre-pregnancy body mass index with outcomes of pregnancy in Chinese women: a retrospective cohort study. PLoS One. 2015;10(8):e0136850. 26313941 10.1371/journal.pone.0136850 PMC4552294

[23] Wen T, Lv Y. Inadequate gestational weight gain and adverse pregnancy outcomes among normal weight women in China. Int J Clin Exp Med. 2015;8(2):2881-6. 25932249 PMC4402896

[24] Wang Y, Mao J, Wang W, et al. Maternal fat free mass during pregnancy is associated with birth weight. Reprod Health. 2017;14(1):47. 28351407 10.1186/s12978-017-0308-3 PMC5371275

[25] Gernand AD, Christian P, Paul RR, et al. Maternal weight and body composition during pregnancy are associated with placental and birth weight in rural Bangladesh. J Nutr. 2012;142(11):2010-6. 22990469 10.3945/jn.112.163634 PMC3498974

[26] Forsum E, Löf M, Olausson H, et al. Maternal body composition in relation to infant birth weight and subcutaneous adipose tissue. Br J Nutr. 2006;96(2):408-14. 16923238 10.1079/bjn20061828

[27] Institute of Medicine, Food and Nutrition Board, National Academy of Sciences, et al. Nutrition during pregnancy. Washington: National Academy Press; 1990. 481 p.

[28] Lederman SA. Variability of water content of lean tissue of pregnant and nonpregnant rats and its effect on body fat estimation. Am J Clin Nutr. 1983;37(4):663-8. 6837497 10.1093/ajcn/37.4.663

[29] Duffus GM, MacGillivray I, Dennis KJ. The relationship between baby weight and changes in maternal weight, total body water, plasma volume, electrolytes and proteins and urinary oestriol excretion. BJOG. 1971;78(2):97-104.

[30] Ghezzi F, Franchi M, Balestreri D, et al. Bioelectrical impedance analysis during pregnancy and neonatal birth weight. Eur J Obstet Gynecol Reprod Biol. 2001;98(2):171-6. 11574127 10.1016/s0301-2115(01)00330-x

[31] Larciprete G, Valensise H, Vasapollo B, et al. Maternal body composition at term gestation and birth weight: is there a link? Acta Diabetol. 2003;40(suppl 1):S222-4. 14618478 10.1007/s00592-003-0071-5

[32] Lederman SA, Paxton A, Heymsfield SB, et al. Maternal body fat and water during pregnancy: do they raise infant birth weight? Am J Obstet Gynecol. 1999;180(1 Pt 1):235-40. 9914610 10.1016/s0002-9378(99)70181-x

[33] Widen EM, Factor-Litvak PR, Gallagher D, et al. The pattern of gestational weight gain is associated with changes in maternal body composition and neonatal size. Matern Child Health J. 2015;19(10):2286-94. 26179720 10.1007/s10995-015-1747-5 PMC4575863

[34] Yip R. Significance of an abnormally low or high hemoglobin concentration during pregnancy: special consideration of iron nutrition. Am J Clin Nutr. 2000;72(1)(suppl):272S-9S. 10871593 10.1093/ajcn/72.1.272S

