# Supplementary figures and images for: Relationship between Maternal Body Composition during Pregnancy and Newborn Birth Weight in Japan
Source: JMA J. 2025 Nov 14;9(1):189–97. doi: 10.31662/jmaj.2025-0060 (PMC12888959; doi:10.31662/jmaj.2025-0060)

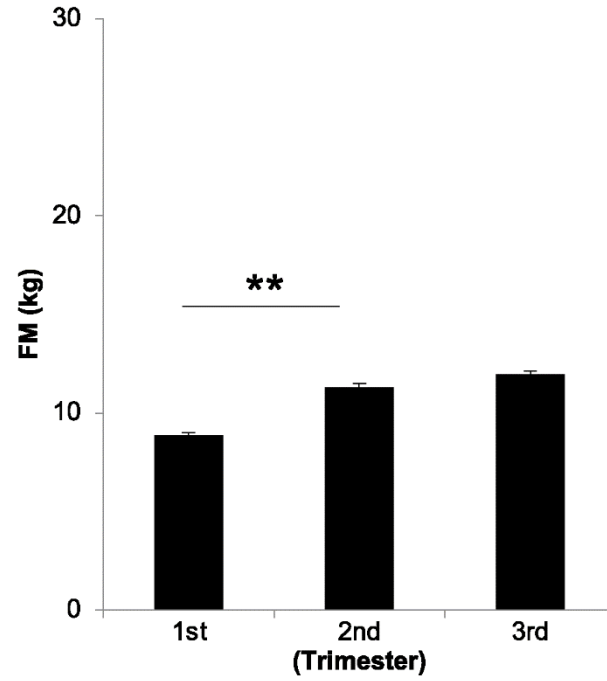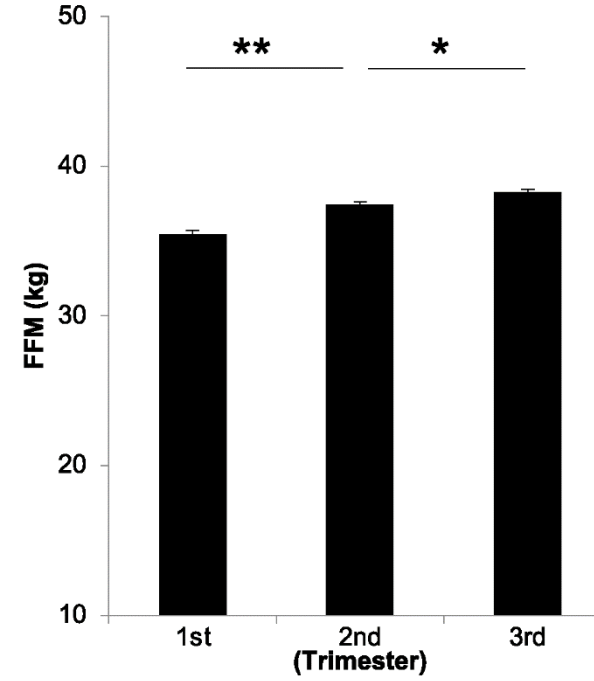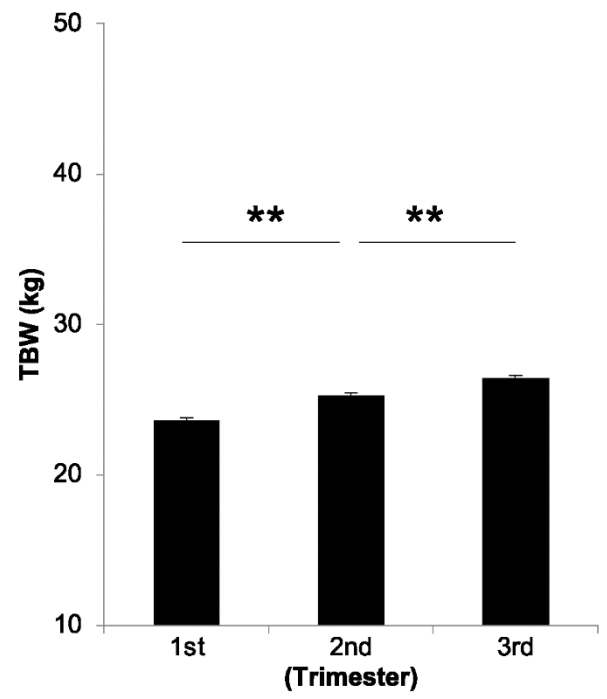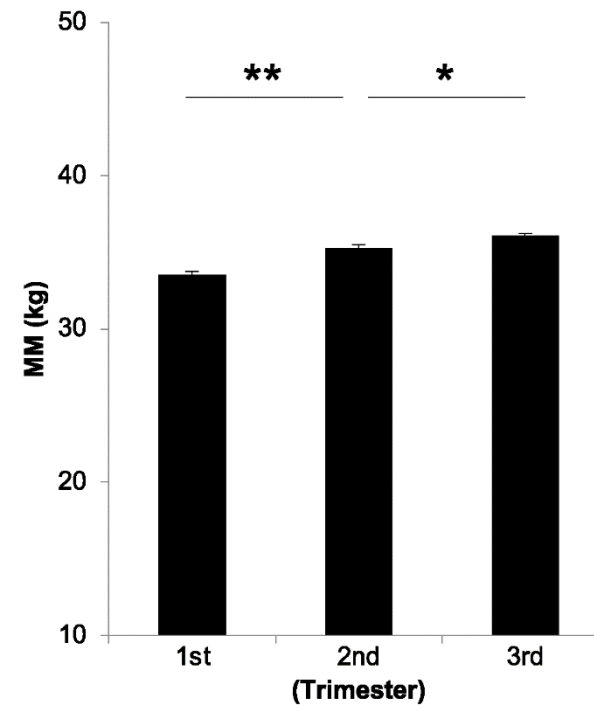

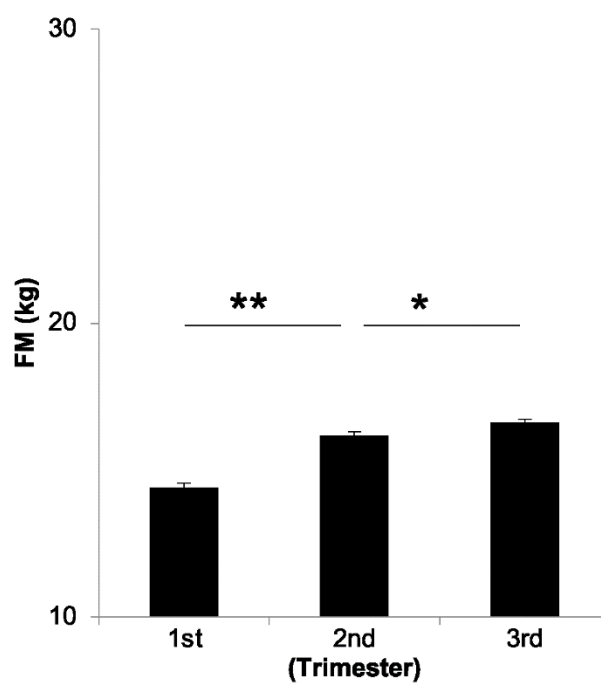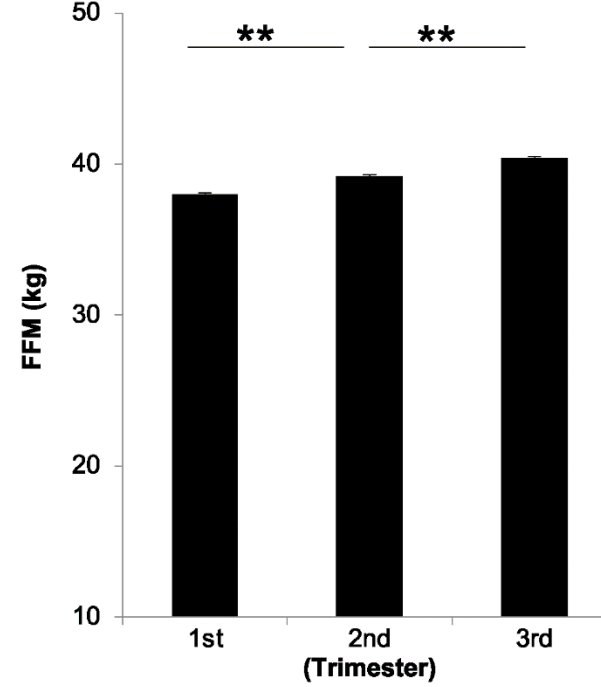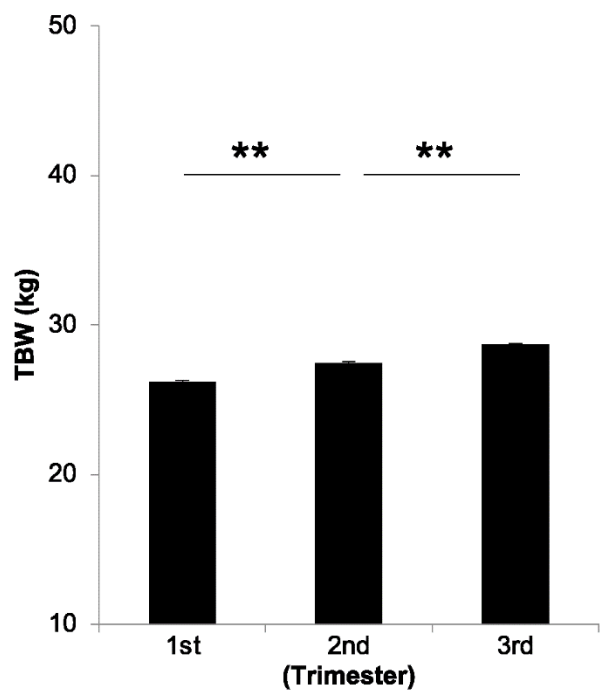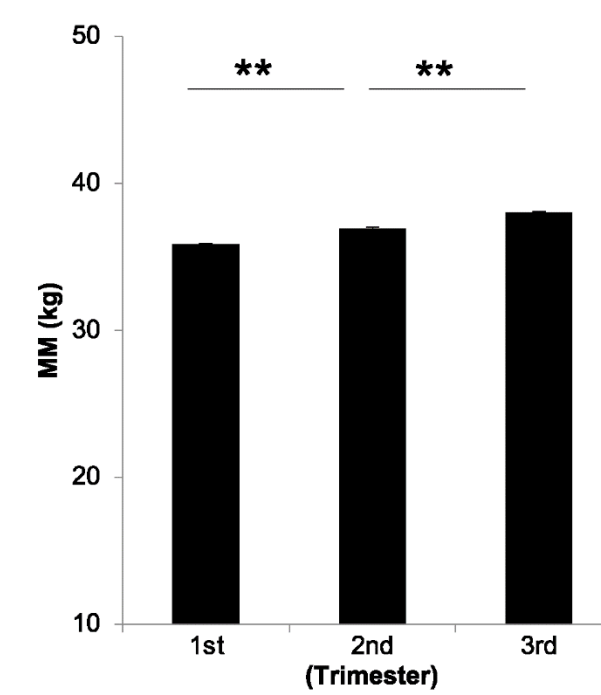

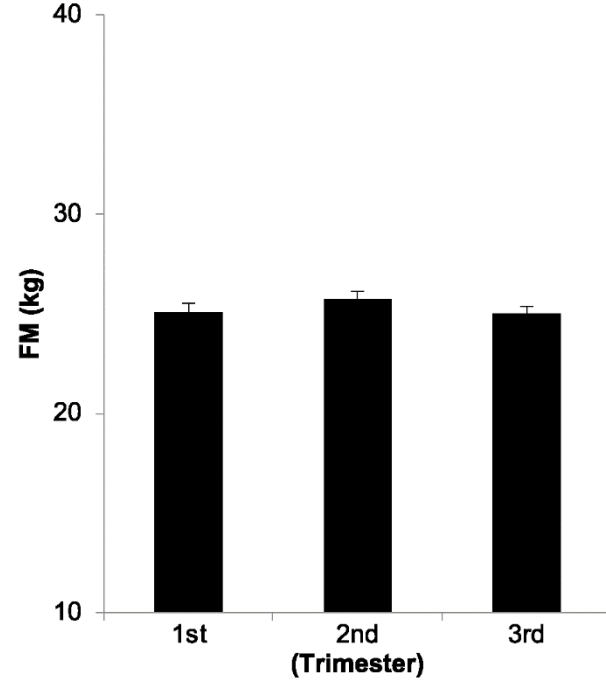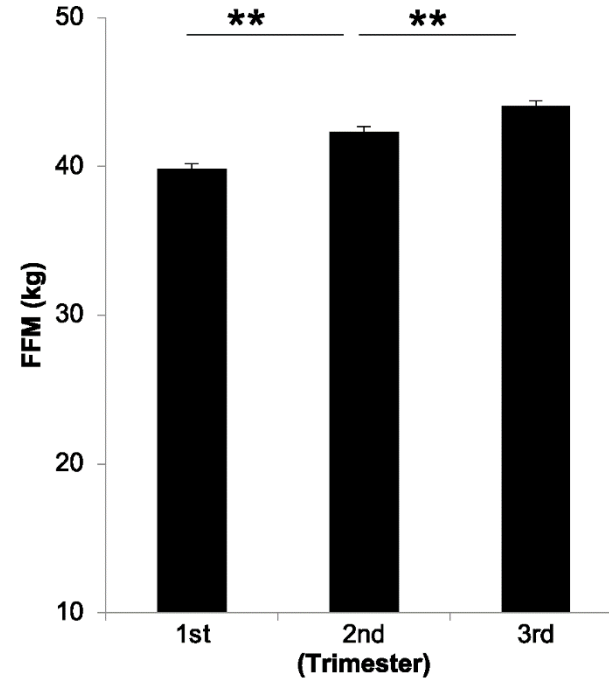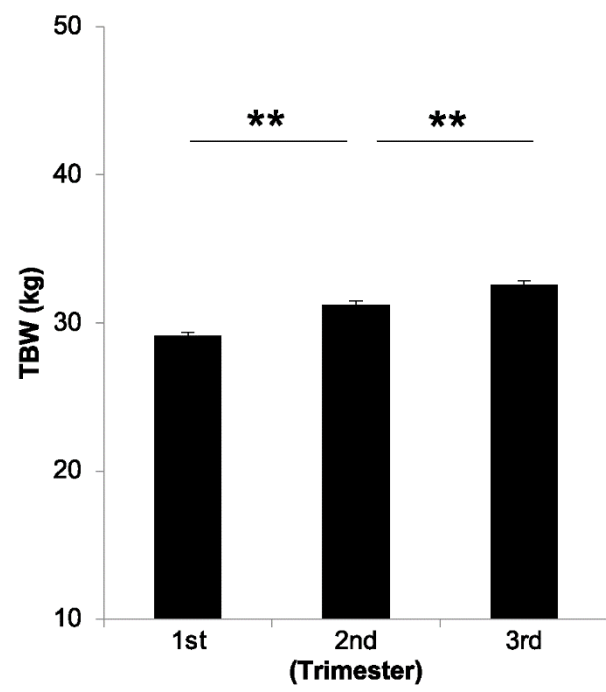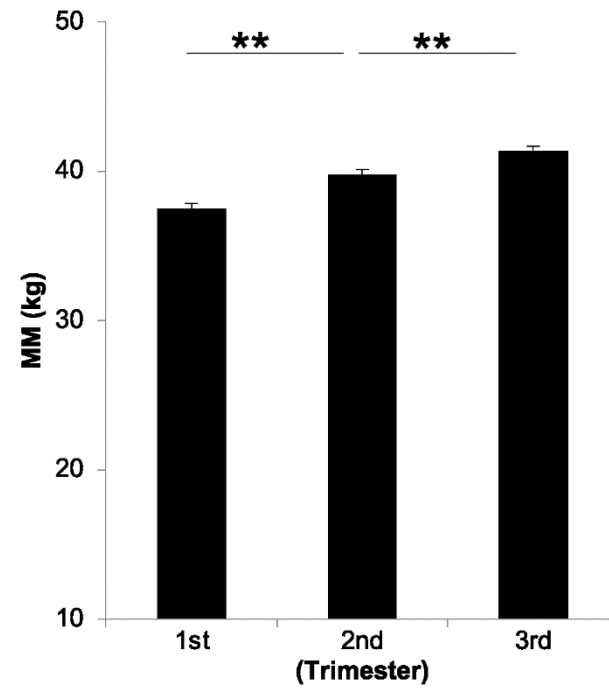

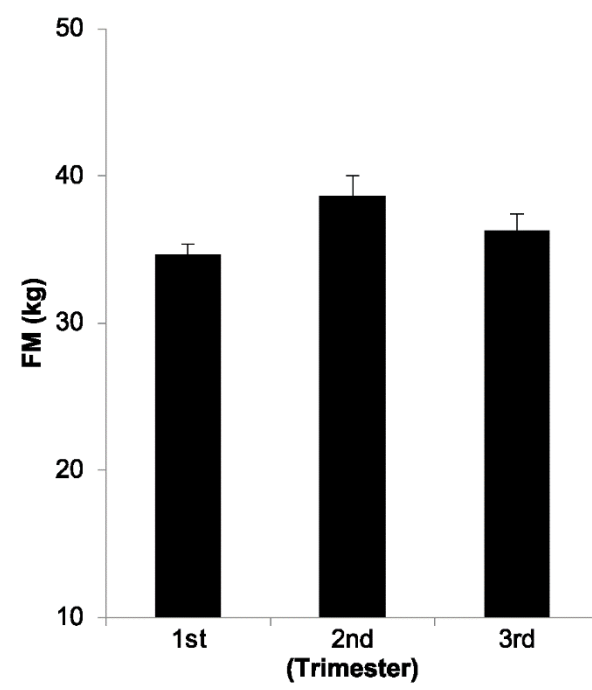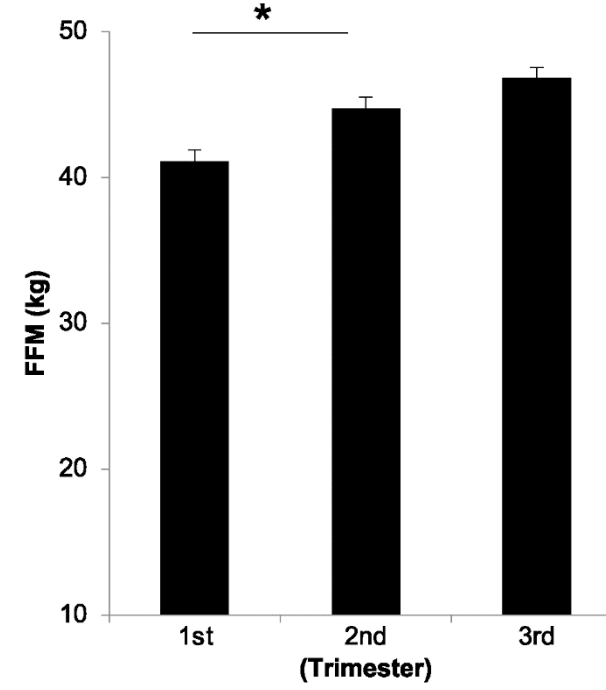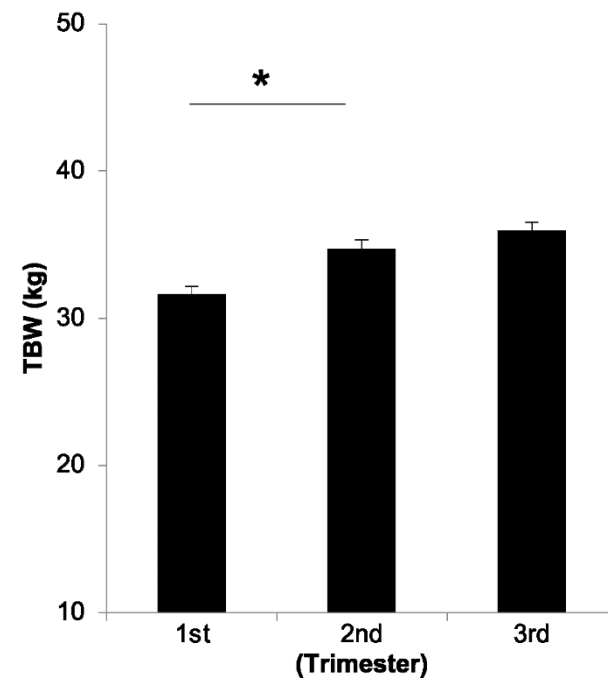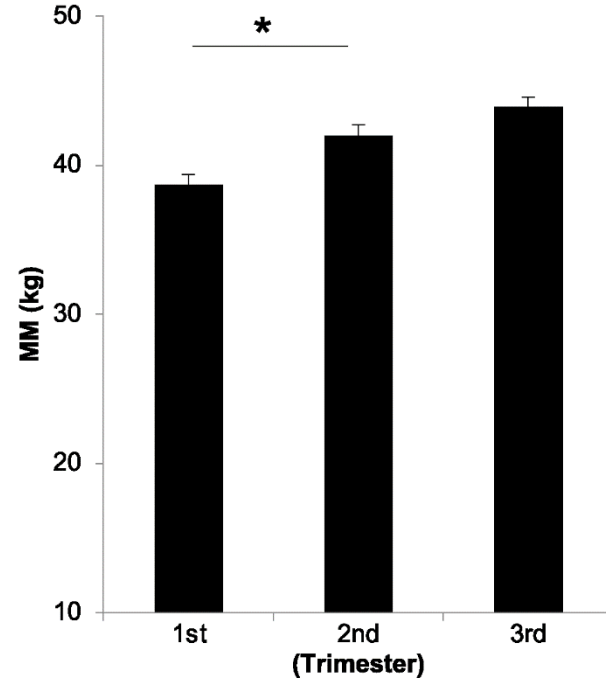

Supplement: Supplementary Material — Supplementary Figure 1. Association between maternal body composition changes and birth weight in women with underweight. FFM: fat-free mass; FM: fat mass; MM: muscle mass TBW: total body water. * p < 0.05, ** p < 0.01 Supplementary Figure 2. Association between maternal body composition changes and birth weight in women with normal weight. FFM: fat-free mass; FM: fat mass; MM: muscle mass TBW: total body water. * p < 0.05, ** p < 0.01 Supplementary Figure 3. Association between maternal body composition changes and birth weight in women with overweight. FFM: fat-free mass; FM: fat mass; MM: muscle mass TBW: total body water. * p < 0.05, ** p < 0.01 Supplementary Figure 4. Association between maternal body composition changes and birth weight in women with obesity. FM FFM: fat-free mass; FM: fat mass; MM: muscle mass TBW: total body water. * p < 0.05, ** p < 0.01 [file 2433-3298-9-1-0189-s001.pdf]
